# Chitosan-Based Nano Systems for Natural Antioxidants in Breast Cancer Therapy

**DOI:** 10.3390/polym15132953

**Published:** 2023-07-05

**Authors:** Yedi Herdiana, Patihul Husni, Siti Nurhasanah, Shaharum Shamsuddin, Nasrul Wathoni

**Affiliations:** 1 Department of Pharmaceutics and Pharmaceutical Technology, Faculty of Pharmacy, Universitas Padjadjaran, Sumedang 45363, Indonesia; patihul.husni@unpad.ac.id (P.H.); nasrul@unpad.ac.id (N.W.); 2 Faculty of Agricultural Industrial Technology, Universitas Padjadjaran, Sumedang 45363, Indonesia; siti.nurhasanah@unpad.ac.id; 3 School of Health Sciences, Universiti Sains Malaysia, Kubang Kerian 16150, Malaysia; shaharum1@usm.my; 4 Nanobiotech Research Initiative, Institute for Research in Molecular Medicine (INFORMM), Universiti Sains Malaysia, Penang 11800, Malaysia; 5 USM-RIKEN Interdisciplinary Collaboration on Advanced Sciences (URICAS), Universiti Sains Malaysia, Penang 11800, Malaysia

**Keywords:** breast cancer, bioavailability, chitosan, oral drug delivery, pharmacokinetic, pharmacodynamic properties

## Abstract

Breast cancer is a major cause of death globally, accounting for around 13% of all deaths. Chemotherapy, the common treatment for cancer, can have side effects that lead to the production of reactive oxygen species (ROS) and an increase in oxidative stress in the body. Antioxidants are important for maintaining the health of cells and helping the immune system function properly. They play a crucial role in balancing the body’s internal environment. Using natural antioxidants is an alternative to mitigate the harmful effects of oxidative stress. However, around 80% of natural antioxidants have limited effectiveness when taken orally because they do not dissolve well in water or other solvents. This poor solubility affects their ability to be absorbed by the body and limits their bioavailability. One strategy that has been considered is to increase their water solubility to increase their oral bioavailability. Chitosan-based nanoparticle (CSNP) systems have been extensively explored due to their reliability and simpler synthesis routes. This review focuses on the various methods of chitosan-based nanoformulation for developing effective oral dosage forms for natural antioxidants based on the pharmacokinetics and pharmacodynamics properties. Chitosan (CS) could be a model, because of its wide use in polymeric NPs research, thus providing a better understanding of the role of vehicles that carry natural antioxidants in maintaining the stability and enhancing the performance of cancer drugs.

## 1. Introduction

Breast cancer is a major health concern affecting women worldwide, with a high incidence rate and significant mortality. In 2020, 2.3 million cases were reported globally, with 685,000 deaths [1,2,3,4]. Breast cancer is the most common cancer and the leading cause of cancer-related deaths among women worldwide. However, its occurrence and characteristics can vary in different countries. Additionally, breast cancer ranks second among all types of cancers, comprising around 12% of total cases [2,5]. However, managing breast cancer presents various difficulties, including assessing risk, predicting diagnostics, preventing metastatic disease, selecting suitable treatments, and ensuring cost-effective approaches [6].

The excessive presence of ROS and an imbalance between ROS and antioxidants in cancer cells can encourage the development of cancer and its progression by triggering genetic mutations and activating cancer-promoting signals [7,8,9,10]. Tumor cells possess robust mechanisms to regulate redox balance and keep oxidative stress at a minimum. However, extreme stress conditions can lead to cell death and contribute to the onset of various illnesses [11,12].

Antioxidants are compounds that can counteract the generation of free radicals and inhibit the process of oxidation [12,13]. They can be classified into two categories based on their source: endogenous antioxidants, derived from enzymes produced within the body, and exogenous antioxidants, obtained from dietary sources such as carotenoids, flavonoids, phenolics, minerals, and vitamins [14]. High intake of antioxidant-rich foods has been linked to a lower risk of cancer [11]. However, the utilization of antioxidant supplements during cancer treatment remains a subject of disagreement among healthcare professionals [12,15,16,17,18]. While some fear it may interfere with the efficacy of chemotherapy, others believe it may allow for higher effective doses and improved tumor response [15]. Breast cancer survivors are particularly likely to use dietary supplements, including antioxidants [13,19]. Antioxidants have traditionally been investigated as antichemo agents for breast carcinogenesis and tumor progression, but there is growing evidence that they may have potential use as additional treatments [12,18]. Extended or prolonged exposure to antioxidants has the potential to confer protection upon women against the onset of breast cancer [11,12,20,21,22]. However, the efficacy and safety of using antioxidants during cancer treatment are not well-established, emphasizing the importance of healthcare providers discussing dietary supplements and antioxidant use with patients undergoing cancer treatment [19].

During drug development, it is crucial to consider factors such as absorption, biodistribution, and metabolism in various tissues [23,24,25]. Pharmacokinetic studies have revealed that natural antioxidants tend to have limited absorption, quick metabolism, and low oral bioavailability [26,27,28,29]. This is exemplified by findings on compounds such as curcumin, a natural antioxidant, which undergoes degradation through processes such as acidic and alkaline hydrolysis, oxidation, and photodecomposition. The degradation rate of curcumin is influenced by pH levels, with a faster degradation rate observed in neutral to basic solutions, while it remains relatively stable at pH levels below 6.5.

Over the past few years, there has been an increasing utilization of nanotechnology in the field of medicine, referred to as nanomedicine, which has had a significant impact [30,31]. Smaller nanoparticles have a higher specific surface area [32]. However, the potential cell toxicity of nanoparticles (NPs) in some situations has raised concerns about their clinical use [30]. Phytochemicals often possess a high molecular weight (MW), limited solubility in water, poor permeability in the gastrointestinal (GI) tract, undergo extensive metabolism before entering the systemic circulation, and exhibit instability in the harsh conditions of the GI environment. The use of nanotechnology has demonstrated promising results in improving the bioavailability of hydrophobic substances such as curcumin [33]. Lately, there has been an increasing fascination with employing bio-based approaches to synthesize NPs, mainly due to their straightforward synthesis process, environmentally friendly nature, stability, and compatibility with biological systems [33,34,35]. Chitosan (CS), a naturally occurring polysaccharide derived from chitin, has gained popularity as a nanocarrier due to its exceptional properties, including biodegradability, compatibility with biological systems, hydrophilic nature, and non-toxicity [31,36,37]. Additionally, CS possesses antimicrobial activity and is abundantly available on Earth [38,39,40,41,42]. CS’s unique properties enable it to interact with various epithelia, making it a potential carrier for drugs targeting these tissues. Chitosan nanoparticles (CSNPs) also protect drugs from external factors such as pH and enzymes and promote controlled and sustained drug release, increasing drug bioavailability [43,44,45]. CSNPs will facilitate the development of novel smart drug-loading materials with potential applications in chemotherapy and physical therapy [46].

Improving the pharmacokinetics and pharmacodynamics of natural antioxidants is crucial in breast cancer therapy [47,48,49]. Oral drug therapy is convenient and ongoing research focuses on identifying new oral drugs and improving the efficacy and safety of existing ones [23,44,50]. As a carrier, chitosan nanoparticles (CSNPs) can be loaded with insoluble drugs, such as quercetin [46]. CS-based formulations have shown promise in enhancing the bioavailability and therapeutic efficacy of natural antioxidants. Recent studies have demonstrated that CS-coated lipid nanocapsules and CSNPs loaded with natural antioxidants can significantly increase their absorption and distribution in the body, leading to improved anti-inflammatory and anticancer activities [51]. To further optimize the delivery of natural antioxidants using CS-based systems, research could focus on exploring different CS derivatives and manufacturing processes to improve their physicochemical properties and interaction with the compounds.

This review addresses the pressing challenge of developing effective oral dosage forms for natural antioxidants, particularly in the context of breast cancer therapy. Excitingly, recent studies have shown promising results for CS-based formulations in enhancing the pharmacokinetics and pharmacodynamics of these compounds. Leveraging the benefits of nanoparticle systems, scientists are actively investigating novel ways to optimize the formulation and delivery of natural antioxidants to maximize their therapeutic potential. As breast cancer remains a major global health concern, with increasing incidence rates and complex management challenges, the development of safe and effective oral drugs is critical for improving patient outcomes. This review offers a comprehensive overview of the latest advances in this field and highlights the urgent need for further research to drive progress toward more effective breast cancer treatment options.

## 2. Antioxidants, Oxidative Stress, and Breast Cancer

### 2.1. Breast Cancer Overview

Breast cancer is a widespread type of cancer that affects women globally, ranking as the second-highest cause of cancer-related deaths [1,2,3,6,52]. However, the occurrence of breast cancer and the number of deaths related to the disease differ in various countries [2]. Breast cancer is a diverse and intricate collection of diseases that exhibit variations in treatment response and prognosis [53,54,55]. Breast cancer can be categorized into four distinct subtypes depending on the presence or absence of estrogen and progesterone hormones, as well as the expression of human epidermal growth factor receptor 2 (HER2):

1. Luminal A breast cancer is characterized by being hormone receptor positive (estrogen receptor and/or progesterone receptor positive) and HER2 negative. These cancers are typically low-grade, have a slow growth rate, and generally have a favorable prognosis.
2. Luminal B breast cancer is hormone receptor positive (estrogen receptor and/or progesterone receptor positive) and can be either HER2 positive or HER2 negative. Luminal B cancers tend to grow slightly faster than the luminal A subtype.
3. Triple-negative/basal-like breast cancer is hormone receptor negative (estrogen receptor and progesterone receptor negative) and HER2 negative. This type of cancer is more prevalent among younger women and African American women. Triple-negative breast cancer (TNBC) is particularly concerning, as over 50% of affected individuals may die within the first six months of developing metastatic disease.
4. HER2-enriched breast cancer is hormone receptor negative (estrogen receptor and progesterone receptor negative) and HER2 positive. HER2-enriched cancers may have a poorer prognosis, but targeted therapies that specifically address the HER2 protein, such as trastuzumab, have proven to be effective treatments.

The occurrence of breast cancer has risen in developed nations, primarily attributed to shifts in environmental and lifestyle factors. In contrast, low- to middle-income countries face challenges in terms of limited resources for preventive screening and appropriate treatment, increasing mortality rates [12,52]. Various risk factors can elevate an individual’s likelihood of developing breast cancer. These encompass genetic mutations, specifically the breast cancer gene 1 (BRCA1) and breast cancer gene 2 (BRCA2) tumor suppressor genes, obesity, exposure to radiation, early onset of menstruation, nulliparity (not having children), late-age childbirth, late-age menopause, alcohol consumption, smoking, and an unhealthy diet. Some of these risk factors, such as age and family history, cannot be modified, while others, such as nulliparity, hormone therapy, obesity, and alcohol consumption, are modifiable lifestyle factors that can be addressed to reduce the risk of breast cancer. In men, risk factors include age, race, genetic mutations, elevated estradiol serum levels, obesity, gynecomastia, history of radiation exposure, diabetes, and orchitis/epididymitis [2,9,12,22,41]. Enhancing health awareness, implementing effective prevention strategies, and ensuring improved access to medical treatment are crucial in mitigating the impact of breast cancer, especially in countries heavily affected by the disease [2]. The decline in breast cancer-related deaths in the United State of America is attributed to improved cancer survival from increased use of adjuvant chemotherapy, rather than advances in prevention or screening [6,12,22,56]. Early-onset breast cancer is more aggressive and alternative splicing plays a significant role in tumor progression, making it a potential therapeutic target.

### 2.2. Oxidative Stress in Breast Cancer

The advancement of cancer is intricately associated with oxidative stress and the production of ROS. This increased ROS production is primarily attributed to an imbalance between oxidants and antioxidants within the cancerous cells [8]. However, when the capacity of antioxidant defense systems is exceeded, oxidative stress occurs, resulting in severe pathophysiological consequences [41]. Elevated levels of ROS can contribute to tumor development and progression by causing gene mutations, activating pro-oncogenic signaling pathways, and directly oxidizing macromolecules. ROS can attack cellular amino acids, lipids, and deoxyribonucleic acid (DNA), leading to cellular damage [16,20,29]. Therefore, understanding the mechanisms underlying redox homeostasis and oxidative stress in tumor cells is crucial for the development of effective cancer therapies [16,29].

Further investigation is necessary to fully understand the complex and multifaceted role of ROS in the development and progression of cancers, including breast cancer [57,58,59,60,61]. The existing evidence linking nonmodifiable risk factors with oxidative stress supports the notion that ROS plays a significant role in initiating, promoting, and advancing breast cancer [20]. This understanding holds the potential to identify novel targets for therapeutic interventions and facilitate the development of more effective treatments for cancer.

In recent decades, a wide range of antioxidants has been developed, some of which demonstrate promise as anticancer drugs [12,22,28,49]. Antioxidant therapeutic approaches encompass enzymatic antioxidants, such as nicotinamide adenine dinucleotide phosphate (NADPH) oxidase inhibitors and SOD mimics, as well as non-enzymatic antioxidants, such as vitamin C, vitamin E, and uric acid [9,13,16]. However, the optimal use of antioxidants in cancer treatment remains a topic of debate, with some studies suggesting that combining multiple antioxidants may yield better long-term results compared to using single antioxidant compounds [62,63].

### 2.3. Antioxidants in Breast Cancer

The role of antioxidants in preventing diseases caused by free radicals has gained much attention in recent times [8,12,17,41]. Antioxidants play a crucial role in scavenging free radicals or ROS and preventing the oxidation of lipids, DNA, sugar, and proteins when present in low concentrations. They are naturally found in plants, and various foods, and are also synthesized within the body [10,26,62]. The consumption of antioxidant-rich vegetables and fruits has been associated with a reduced risk of diseases caused by ROS, including cardiovascular diseases and cancer [6,26,64]. Natural antioxidants such as polyphenols and carotenoids exhibit beneficial properties such as anti-atherosclerosis, anti-aging, anti-inflammatory, and anticancer effects. By safeguarding tissues and skin against damage caused by free radicals, antioxidants contribute to promoting overall health and well-being, particularly as individuals age [49].

The utilization of antioxidant supplements among cancer patients has been estimated to range from 13% to 87% based on various studies [12,15,22,65]. While higher levels of natural antioxidants within the body may offer protection against chemotherapy-induced oxidative stress, certain anticancer medications function by generating free radicals, which cause cellular damage and induce necrosis in cancerous cells. As a result, the use of higher-than-dietary doses of antioxidants as a potential primary or secondary strategy for preventing cancer is a topic of interest. However, the variation in the percentage range can be attributed to factors such as the type of cancer, age, educational background, complementary medicine usage, and ethnicity within the study population [13].

Antioxidants can be broadly categorized into three main classes

1. The primary defense system of antioxidants comprises enzymes such as SOD, glutathione reductase (GR), catalase (CAT), and essential minerals such as zinc, selenium, and copper.
2. The secondary defense system of antioxidants includes molecules such as glutathione (GSH), flavonoids, carotenoids, vitamin C, and vitamin E.
3. The tertiary defense system of antioxidants involves a complex combination of chemicals responsible for repairing damaged DNA, proteins, oxidized lipids, and peroxides. Examples of these include DNA repair enzymes, methionine sulphoxide reductase, proteases, lipases, transferases, and other related substances [10].

Studies have shown that taking antioxidants during chemotherapy or radiation therapy may worsen breast cancer prognosis in postmenopausal women. Therefore, it is recommended to avoid using antioxidants during these treatments [19]. A small study found that taking antioxidant supplements increased the risk of cancer recurrence and death. However, taking a regular multivitamin did not have any effect on the risk [56]. Breast cancer survivors often turn to complementary and alternative medicines more than other cancer patients or healthy individuals. The most commonly used form of complementary and alternative medicine among them is dietary supplements, including herbal remedies [19]. Cancer patients, in general, widely use CAM supplements, with dietary supplements, vitamins, minerals, herbal remedies, and antioxidants being especially popular choices [18].

Dietary antioxidants derived from natural sources have demonstrated the ability to inhibit both early and late stages of cancer development, making them potential therapeutic agents for breast cancer. Clinical trials have investigated the use of antioxidant supplementation either as a standalone therapy or as an adjunct therapy, as well as for alleviating side effects associated with cancer treatments [12].

Antioxidants may not effectively prevent cancer due to several factors, including the use of pharmacological instead of dietary doses in studies, uneven distribution in tissues, and potential pro-oxidant effects. Chemotherapy drugs generate high levels of ROS, and taking antioxidants during treatment may hinder their effectiveness. Weak pro-oxidants may be a more promising approach to boosting antioxidant activity in cancer patients, but further research is needed to understand the biological rationale and long-term effects of interventions. Understanding these mechanisms could lead to the development of new cancer prevention and treatment methods.

The role of the nuclear factor erythroid 2–related factor 2 (Nrf2) is associated with improving antioxidant properties, protecting specific genes, and regulating resistance to chemotherapy by activating genes involved in glutathione synthesis. The Nrf2 is involved in breast cancer development, angiogenesis, stem cell generation, metastasis, and effective treatment strategies for breast cancer cases with abnormal Nrf2 expression. Nrf2 is crucial for maintaining cellular redox homeostasis by controlling the expression of GSH, thioredoxin (TXN), and NADPH to manage the level of reactive oxygen species (ROS). Gaining a better understanding of Nrf2-associated events has the potential to lead to improved treatment options for breast cancer [5].

Certain anticancer agents possess antioxidant properties alongside their primary anticancer effects [66,67,68,69,70,71,72]. For instance, various natural compounds derived from fruits and vegetables, including curcumin, resveratrol, epigallocatechin-3-gallate (EGCG), vitamin E, lycopene, apigenin (API), and baicalein (BCL) [72], exhibit both anticancer and antioxidant properties. These compounds can help safeguard cells against oxidative stress and may offer additional health benefits, such as reducing inflammation and enhancing immune function. Natural anticancer drugs are often cost-effective, have multiple mechanisms of action, and can be effective against chemotherapy-resistant cancer cells. However, the precise mechanisms through which flavones exert their anticancer effects are not fully understood. Nevertheless, it has been established that they can impede cancer cell proliferation, induce apoptosis, influence differentiation, inhibit angiogenesis and inflammation, and suppress metastasis [72].

Recent research has highlighted the potential of natural antioxidants in breast cancer treatment. Compounds such as curcumin, resveratrol, EGCG from green tea, lycopene, quercetin, and vitamins A, C, and E have shown anticancer properties [73]. Medicinal plants have long been used globally for disease treatment, with many modern drugs derived from plant sources and their derivatives. Notably, Hibiscus sabdariffa has medicinal properties [74]. Natural plant extracts have demonstrated promising anti-tumor responses and improved pharmacological activity with reduced toxicity in advanced breast cancer patients. Examples include compounds from Curcuma longa, Piper longum, Nigella sativa, Murrayakoenigii, Amora rohituka, Withania somnifera, and Dimocarpus longan, exhibiting anticancer activity, particularly against breast cancer [75]. These medicinal plants and their derivatives contribute significantly to the development of anticancer drugs, accounting for about 70% of anticancer compounds. Flavonoids, such as flavones, flavanones, flavanols, flavonols, isoflavones, and anthocyanidins, found in plants, along with carotenoids, polyphenols, and other antioxidant compounds, are being explored for their potential in cancer therapy. Incorporating functional foods with high antioxidant potential offers effective and affordable management of free-radical-related diseases while avoiding the toxicities and side effects associated with conventional medications. The use of natural antioxidants shows promise for breast cancer treatment, providing benefits in terms of efficacy, reduced toxicity, and cost-effectiveness. Further research is needed to uncover their mechanisms of action and optimize their application in clinical settings [76].

### 2.4. Target Action of Antioxidants in Breast Cancer

Antioxidants can target several key pathways and mechanisms involved in the development, progression, and treatment of breast cancer [9,47,56]. These targets include ROS, apoptosis, inflammation, chemotherapy and radiotherapy side effects, hormone receptor signaling, and gene expression. Antioxidants can scavenge ROS and reduce oxidative stress in breast cancer cells [77,78,79], modulate the apoptotic pathways to inhibit tumor growth, reduce inflammation in breast cancer tissues, protect normal cells from oxidative damage caused by chemotherapy and radiotherapy, modulate hormone receptor signaling, and alter gene expression in breast cancer cells [12,13,47,80]. However, further research is needed to determine the optimal use of antioxidants in breast cancer prevention and treatment, as some studies have raised concerns about potential pro-tumor effects at high doses.

The target of antioxidants in breast cancer can be located within the cancer cells themselves (intracellular) or within the microenvironment surrounding the cancer cells (extracellular) [81]. However, the boundaries between intracellular and extracellular targets are not always clear-cut, and many targets can have both intracellular and extracellular effects. Targeting both types of targets can modify the microenvironment to be less supportive of tumor growth, which is important in improving patient outcomes. The target of antioxidants is divided into two parts:

1. Intracellular targets: these targets involve mechanisms and pathways that are primarily located within the cancer cells themselves.
  (a) ROS;
  (b) Apoptosis;
  (c) Hormone receptor signaling;
  (d) Gene expression.
2. Extracellular targets: these targets involve the microenvironment surrounding the cancer cells, including the extracellular matrix, stromal cells, and immune cells.
  (a) Inflammation;
  (b) Chemotherapy and radiotherapy side effects.

The ideal design for antioxidant delivery depends on the specific target, whether intracellular or extracellular. Intracellular targets require delivery directly to cancer cells through NPs or carrier systems. Extracellular targets can be addressed through systemic administration or targeted delivery to specific molecules or cells in the microenvironment. The design should consider the unique characteristics of the cancer and its microenvironment [82].

### 2.5. Strategies to Improve the Delivery of Antioxidants Using Chitosan

Despite some progress, antioxidant therapies have generally fallen short of the initial expectations. Several factors could contribute to the limited effectiveness of antioxidants, including inadequate dosing and timing of antioxidant supplementation, suboptimal bioavailability, diverse microenvironments where antioxidants need to exert their action, variations in the antioxidant status of the study population, and the non-targeted delivery of antioxidant compounds. Concerning dosage, some studies may have administered insufficient amounts of antioxidants to evaluate their potential benefits, while excessive concentrations could have resulted in pro-oxidant effects in other cases, raising safety concerns [82].

The oral route is the most convenient and preferable method of administration for maximum patient compliance, particularly in cancer chemotherapy. Achieving oral administration of anticancer drugs would greatly enhance the convenience of treatment for patients [83,84,85]. However, there are significant challenges to overcome when it comes to effectively delivering orally administered antioxidants. One such challenge is the need to solubilize antioxidants in the GI fluids, which have varying pH levels and contain enzymes capable of breaking down these compounds. As a result, the oral bioavailability of these drugs can be significantly reduced. Additionally, the lipophilic nature of phytochemical antioxidants often poses an additional barrier to their absorption in the intestines. Hence, the primary factors contributing to the limited oral bioavailability of antioxidants include their high lipophilicity, instability in GI fluids, and poor absorption in the intestines [51].

The process of drug absorption through biological membranes, particularly the GI wall, is intricate and influenced by various factors. The chemical and biological conditions within the GI tract, including pH levels and microbial flora, play a significant role in drug absorption. For a drug to attain adequate oral bioavailability, it must possess stability to withstand the harsh GI environment and exhibit specific physicochemical properties that enable it to permeate the GI wall [86,87]. Lipinski’s rule, a predictive model, suggests that molecules with an MW not exceeding 500 daltons, no more than five hydrogen bond donors, no more than 10 hydrogen bond acceptors, and a calculated partition coefficient (LogP) no higher than five, are more likely to be optimally absorbed in the GI tract. Nevertheless, there are exceptions to this rule for molecules that are absorbed via specific transporters. Additionally, adjuvants such as food or other drugs can significantly influence the bioavailability of certain compounds by affecting their bioaccessibility and transport across GI tract membranes [26].

**Figure 1:**
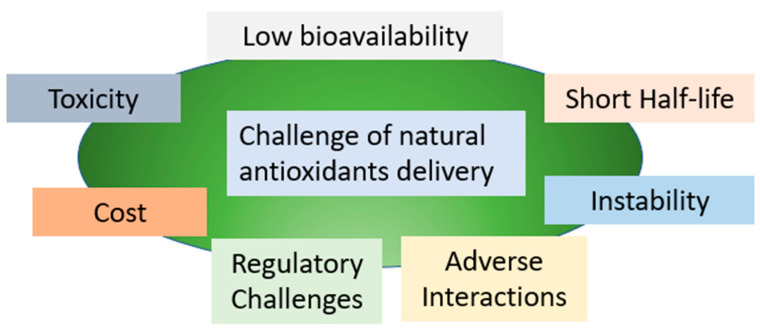
The challenge of natural antioxidants delivery.

The delivery of natural antioxidants can be challenging due to several factors, as shown in Figure 1. To overcome these challenges, researchers and industry professionals are exploring innovative approaches to enhance the bioavailability, stability, and efficacy of natural antioxidants. These include the use of novel delivery systems, formulation optimization, and quality control measures to ensure product safety and efficacy. Additionally, a better understanding of the underlying mechanisms of natural antioxidants and their interactions with other compounds in the body can help to optimize their therapeutic potential and minimize potential toxicity.

The ideal vehicle for natural antioxidants should have the following characteristics:

1. Biocompatibility and Biodegradability: The vehicle should be non-toxic, non-immunogenic, and non-inflammatory, and should not elicit any adverse reactions or toxicity in the body.
2. Enhanced Stability: The vehicle should protect the antioxidants from degradation and enhance their stability, particularly during storage and transportation, and be acid tolerant, and GI enzyme stable.
3. Controlled Release: The vehicle should allow for a controlled release of antioxidants over a specific period, thereby enhancing their efficacy and duration of action.
4. Optimal Bioavailability: The vehicle should improve the absorption and bioavailability of antioxidants, especially in the gastrointestinal (GI) tract, and extend the duration of contact between the drug and the mucosa to enhance drug absorption.
5. Targeted Delivery: The vehicle should be designed to target specific sites in the body where antioxidants are required, thereby reducing systemic toxicity and enhancing therapeutic efficacy.
6. Ease of Administration: The vehicle should be easy to administer, preferably orally, and should not require specialized equipment or expertise.
7. Cost-Effective: The vehicle should be cost-effective and scalable, thereby enabling its widespread use and accessibility.

CS is a versatile compound with unique properties that make it an attractive drug carrier for delivering antioxidants in cancer treatment [34,50,51]. One such antioxidant mixture, PFM, containing curcumin, resveratrol, EGCG, and quercetin, has shown potential for exerting anticancer effects. Chemical modifications of CS, such as N-acylation, have been used to enhance its hydrophobic properties and form amphiphilic NPs for drug delivery [41,88,89]. In addition, electrostatic-interaction-based complexes of CS with negatively charged molecules, physical modification of CS, and creating temperature, pH, ultrasound, and magnetic-sensitive polymers have been utilized to effectively deliver drugs [36,38,45,90,91].

Modifying the surface of CS can help to deliver drugs directly to specific cells. By changing the surface of CS, it can bind to target cell receptors and enter the cells through a special pathway called endocytosis [88,89,90,92]. For example, carboxymethyl-modified CS and N-trimethyl chitosan (TMC) (N-TMC), a quaternized derivative of CS, have been used to improve solubility and aqueous solubility over a wide range of pH values [38,91]. These modifications of CS have been shown to improve drug delivery and increase the effectiveness of cancer treatment. The use of CS in drug delivery systems offers a promising avenue for developing more effective cancer therapies [27,36,45,91].

## 3. Delivery of Antioxidants Using Chitosan Nanoparticles

Nanomaterials derived from CS or its derivatives are extensively studied as nanocarriers with a high potential. This is attributed to their desirable characteristics, including biodegradability, excellent biocompatibility, non-toxicity, low immunogenicity, versatile properties, and advantageous biological effects [93]. Small-sized CSNPs offer significant benefits in enhancing the bioactivity of encapsulated phytochemicals through improved pharmacokinetics, targeted delivery, and reduced toxicity [45]. Furthermore, the particle size (PS), the polydispersity index (PDI), and the zeta potential (ZP) of CSNPs have a significant impact on the delivery of phytochemicals. The particle size, which is affected by the concentration of CS, should be carefully adjusted to achieve optimal oral delivery. A lower PDI signifies better uniformity and colloidal stability, essential for efficient phytochemical-loaded CSNPs. Various parameters, such as CS’s characteristics and crosslinking agent concentration, affect CS-NP formation. Considering these factors ensures the development of CSNPs with optimal PS, low PDI, and suitable ZP, enhancing the overall performance and therapeutic efficacy of phytochemical-loaded CSNPs [51].

To create chitosan nanoparticles (CSNPs), several factors are important, including the concentration of chitosan, its molecular weight (MW), degree of deacetylation (DD), crosslinking agent concentration, and the zeta potential (ZP). When chitosan has a higher MW, it becomes more viscous and leads to larger particle sizes. To obtain smaller-sized nanoparticles, it is advisable to use chitosan with lower or medium MW at lower concentrations. Smaller nanoparticles not only improve drug release but also have positive zeta potential values and high loading efficiency. Generally, a higher drug concentration improves the loading efficiency until it reaches the maximum charge capacity [91,93].

### 3.1. Preparation of Chitosan-Based Nanoparticles

**Figure 2:**
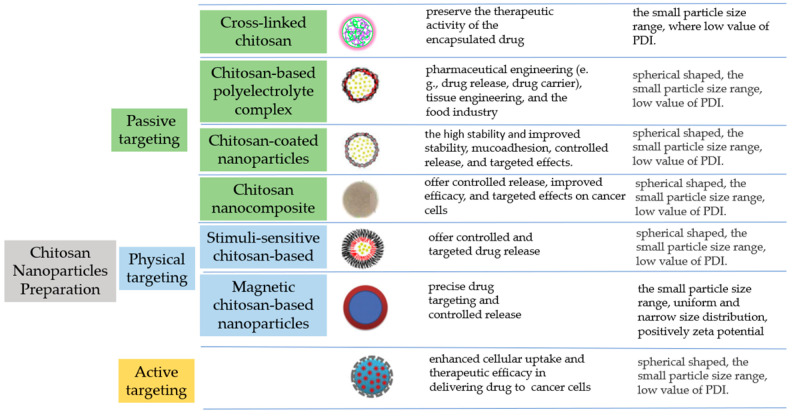
Strategies to improve the delivery of antioxidants using chitosan.

The characteristics and stability of CSNPs emphasize their small particle size, low PDI, and high ZP, which contribute to their stability and mucoadhesive properties. The passage also mentions the need for further studies to establish the bioavailability of phytochemicals and highlights the importance of evaluating the oral bioavailability of different classes of plant-derived antioxidants, including carotenoids/apocarotenoids, polyphenolics, and organosulfur compounds. The strategy for increasing the delivery of antioxidants using CS can be described as follows (Figure 2).

#### 3.1.1. Passive Targeting

1. Crosslinked chitosan NPs

Covalent crosslinking is a commonly employed technique to produce nanoscale particles using CS and its derivatives. This method involves the formation of covalent bonds between CS chains and crosslinkers. Crosslinking agents such as tripolyphosphate (TPP), dicarboxylic acid, glutaraldehyde, and epichlorohydrin are widely utilized. CSNPs prepared through this approach exhibit effective loading capacities for both hydrophilic and hydrophobic drugs, with loading efficiencies influenced by the drug’s characteristics and the specific preparation method. The ionic gelation method is extensively employed for nanoparticle fabrication due to its ability to maintain the therapeutic activity of the encapsulated drug [45,89,93].

2. Chitosan-based polyelectrolyte complex NPs

NPs prepared through electrostatic interactions between oppositely charged polyions have gained significant attention in drug delivery [94]. The formation and characteristics of these complexes are influenced by various factors, including the ionization rate, charge distribution, chain flexibility, ionic strength, polymer concentration, pH, temperature, and reaction time of CS [95]. Despite being influenced by multiple factors, the fabrication of these NPs is uncomplicated and can be carried out under mild conditions, eliminating the requirement for harmful organic reagents [96].

3. Chitosan-coated nanoparticles

CS-coated NPs have gained attention in drug delivery due to their advantages, including improved stability, mucoadhesion, controlled release, and targeted effects [97,98]. The positive surface charge and hydrogen bonding of CS enhance cellular uptake. Combining CS with other nanocarriers expands its benefits, such as pH-responsive release and increased bioavailability. These versatile NPs have the potential to revolutionize drug delivery by improving stability, enabling targeted delivery, and enhancing therapeutic outcomes in various applications.

4. Chitosan nanocomposite

CS derivative nanocomposites have gained significant attention due to their unique physical and chemical properties. The presence of amine (NH_2_) and hydroxyl (OH) surface groups facilitates the formation of various intermolecular and intramolecular hydrogen bonds, making it possible to incorporate NPs as fillers. CS, known for being eco-friendly, cost-effective, sustainable, and renewable, has been extensively explored as a matrix for nanocomposites. Researchers have focused on studying the chemical structure, shaping processes, properties, and applications of CS-based nanocomposites [99].

#### 3.1.2. Physical Targeting

1. Stimuli-sensitive chitosan-based nanoparticles

Stimuli-responsive polymers, such as thermo-responsive CSNPs, are being utilized in smart drug delivery systems to achieve controlled and targeted drug release. Smart drug delivery systems use special materials that respond to certain triggers such as temperature or pH to release drugs in a controlled way. This can improve treatment and reduce side effects. These systems are being improved for better drug delivery in the future [100].

2. Magnetic chitosan-based nanoparticles

A magnetic nanoparticle is usually composed of magnetic cores and a polymeric shell having favorable functional groups and features for various applications [101]. These particles possess the ability to be directed to specific locations within the body through the application of a magnetic field, enabling precise and targeted drug delivery. Moreover, drug release from these NPs can be triggered by electromagnetic waves. Notably, these particles exhibit low toxicity and are biodegradable, further enhancing their attractiveness for biomedical applications [101].

#### 3.1.3. Active Targeting

NPs are valuable tools for targeted drug delivery in cancer therapy. Ligand–receptor interactions play a crucial role in achieving specific delivery. These targeted NPs show enhanced cellular uptake and therapeutic efficacy in delivering drugs to cancer cells. Ligand–receptor-based nanoparticle systems have the potential for selective and efficient drug delivery in cancer therapy [36].

Chitosan nanoparticles can be prepared using various methods, including ionic gelation, microemulsion, emulsion-based solvent evaporation, and emulsification solvent diffusion [102]. Among these methods, the ionic gelation technique stands out as an attractive choice for chitosan nanoparticle preparation [103]. Researchers have extensively studied chitosan nanoparticles and developed different methods considering factors such as size, stability, drug loading capacity, and retention time [104].

In addition to nanoparticles, various other types of chitosan derivatives, such as sponges, films, hydrogels, and scaffolds, are utilized to encapsulate and deliver drugs, thereby facilitating improved drug bioavailability [96,104]. Chitosan nanoparticles offer high drug loading and controlled release, while chitosan microparticles provide sustained release and drug protection. Further modifications, such as incorporating other materials, can enhance their functionality and performance based on specific drug delivery requirements. Chitosan derivatives are primarily obtained through the modification of hydroxyl and amino groups in the chitosan structure. By decorating chitosan with different functional groups, desired products with excellent properties can be achieved, contributing to its extensive use in the biomedical field [105].

### 3.2. Pharmacokinetic Properties Enhancement Delivery of Antioxidants Using Chitosan

The enhancement of the oral pharmacokinetic properties of drugs is crucial for maximizing their activity and therapeutic efficacy [93,106,107]. Pharmacokinetics refers to the study of drug absorption, distribution, metabolism, and elimination within the body [25,108]. When it comes to oral administration, optimizing these parameters is of the utmost importance due to the challenges associated with oral drug delivery [109]. Enhancing the oral pharmacokinetic properties can significantly impact drug activity by improving their bioavailability, distribution to target tissues, and overall systemic exposure. These enhancements directly impact drug activity, leading to improved therapeutic efficacy, reduced side effects, and better patient outcomes in oral drug administration [93].

#### 3.2.1. Absorption

One key aspect of enhancing oral pharmacokinetic properties is improving drug solubility and dissolution. Many drugs exhibit poor solubility in aqueous environments, leading to limited absorption and reduced bioavailability. NPs offer a solution by providing a high surface-area-to-volume ratio, enabling efficient drug dispersion and dissolution. By formulating drugs into NPs, their solubility can be enhanced, resulting in improved oral absorption and increased bioavailability. This enhancement in solubility directly translates to increased drug activity and effectiveness. It provides the benefit of delivering a concentrated amount of drugs directly to the absorption membrane, which serves as a driving force for the passive uptake of drugs [110]. The passage of these drugs between the cells is limited by the tight junction proteins that serve as a fencing mechanism, thus regulating the paracellular epithelial permeability.

CSNPs play a significant role in enhancing the absorption of drugs in oral delivery by leveraging their unique properties. The mechanism of action involves several factors that contribute to improved drug absorption:

- Protection and stabilization: CSNPs such as enteric coatings can shield drugs from degradation within the harsh acidic conditions of the stomach. The NPs act as a protective barrier, preventing the drug from premature degradation and ensuring its integrity until it reaches the absorption site. This protection enables a higher concentration of the active drug to be available for absorption, thereby enhancing the overall absorption efficiency [110].
- Mucoadhesion: CSNPs exhibit mucoadhesive characteristics, enabling them to adhere to the mucosal surfaces of the GI tract. Through the interaction between the positively charged surface of CS and the negatively charged mucosal surfaces, these NPs promote extended contact between the NPs and the site of absorption. This extended contact enhances drug absorption by increasing the residence time and promoting closer interaction between the drug-loaded NPs and the underlying tissues.
- Permeability enhancement: CSNPs can enhance the permeability of drugs across the mucosal barriers of the GI tract. The presence of CS in the nanoparticle formulation can open up tight junctions between the epithelial cells, temporarily increasing the paracellular transport of drugs. This opening of tight junctions allows for improved drug diffusion and absorption through the intercellular spaces, leading to enhanced bioavailability [110].
- Increased surface area: CSNPs have a high surface-area-to-volume ratio due to their small particle size. This increased surface area provides more contact points between the drug-loaded NPs and the absorption site, facilitating efficient drug absorption. The larger surface area allows for greater interaction with the absorptive surfaces, increasing the chances of drug molecules being taken up into the systemic circulation [106].
- Modulation of drug release: CSNPs can be designed to achieve controlled and sustained drug release profiles. By encapsulating drugs within the NPs, their release can be modified and extended over time. This controlled release pattern ensures a gradual and consistent availability of the drug at the absorption site, optimizing the absorption efficiency and reducing the potential for dose dumping [33,51,100].
- Targeted release: The release of the payload from these nanocarriers is selectively activated by the presence of intestinal alkaline phosphatase (IAP), an enzyme located on the cell membrane. These virus-mimicking nanocarriers possess a surface with a high density of anionic and cationic charges, allowing them to penetrate the mucus gel layer and achieve targeted release of their cargo directly at the epithelial cells [110].
- Efflux inhibition: This refers to the process of blocking or reducing the activity of efflux transporters, which are proteins that pump substances out of cells. By inhibiting these transporters, the absorption of certain substances can be increased [111].

These mechanisms collectively improve drug bioavailability and absorption efficiency, offering potential advantages for oral drug delivery and improving therapeutic outcomes. The absorption of NPs through the epithelial cells or M cells following oral administration is significantly influenced by the size of the NPs. The size of the NPs affects both the efficiency and speed of oral absorption. Smaller NPs are primarily taken up by intestinal cells, while larger NPs are mainly absorbed by M cells. Gastro-retentive drug delivery systems (GRDDS) are designed to enhance the release efficiency of drugs by prolonging their residence time in the stomach. This approach aims to optimize the bioavailability of drugs, especially those with limited absorption windows in the GI tract. Floating tablets are an example of GRDDS, as they can extend the gastro-retention time and improve the bioavailability of such drugs [112].

#### 3.2.2. Distribution

The distribution of drugs within the body is a crucial factor in determining their therapeutic efficacy. CSNPs in oral delivery systems can impact drugs’ distributions through various mechanisms, enhancing their effectiveness at the target site while minimizing exposure to non-target tissues [106].

One mechanism by which CSNPs improve drug distribution is through their ability to encapsulate drugs and protect them from degradation [100]. By encapsulating drugs within NPs, the stability of the drug is increased, preventing premature degradation in the GI tract. This protection ensures that a higher concentration of the active drug reaches the systemic circulation, allowing for improved distribution to target tissues [23,106].

Moreover, CSNPs can be designed to exhibit specific surface properties that promote targeted delivery to desired sites within the body. Surface modifications and functionalization of NPs enable active targeting, allowing them to selectively accumulate in specific tissues or cells. Ligands or targeting moieties can be attached to the nanoparticle surface, which interacts with specific receptors or markers present in the target tissues. This active targeting approach enhances drug distribution to the desired site, maximizing the drug’s therapeutic effect while reducing exposure to non-target tissues. This targeted drug delivery strategy improves drug distribution efficiency and can potentially enhance treatment outcomes [36].

CSNPs offer passive targeting capabilities in addition to active targeting. Their small size enables effective tissue penetration, reaching sites inaccessible to larger drug molecules. The positively charged surfaces of CSNPs interact with cell membranes or extracellular matrices, facilitating accumulation in specific tissues or cells. This passive targeting mechanism improves drug distribution and concentration at the target site, enhancing therapeutic efficacy [81,82,100].

#### 3.2.3. Metabolism

The metabolism and elimination of drugs also play a crucial role in their overall activity and therapeutic duration. NPs can impact drug metabolism by modulating the activity of drug-metabolizing enzymes and transporters in the liver and other organs [113,114]. By improving drug metabolism and elimination, nanoparticle-based delivery systems contribute to the maintenance of optimal drug levels, prolonged drug activity, and improved therapeutic outcomes [115]. These NPs can influence the expression and function of enzymes such as cytochrome P450 (CYP) enzymes, which are responsible for the metabolism of many drugs. CSNPs have been shown to inhibit certain CYP enzymes, leading to decreased drug metabolism and increased systemic exposure to the active drug [116,117].

These NPs have been reported to inhibit the activity of efflux transporters such as P-glycoprotein (P-gp), which play a role in limiting drug absorption and promoting drug elimination. By inhibiting these transporters, CSNPs can increase drug absorption and reduce drug efflux, leading to higher systemic exposure and enhanced drug activity [111,118].

#### 3.2.4. Excretion

CSNPs used in oral drug delivery systems have a positive impact on drug excretion, leading to enhanced drug activity. These NPs modulate drug transporters, which are responsible for the movement of drugs across cell membranes. By influencing these transporters, CSNPs can affect drug absorption and excretion, ultimately enhancing drug activity [119,120,121].

Furthermore, CSNPs contribute to the enhancement of renal clearance. Their small size and favorable surface properties allow them to be efficiently filtered by the kidneys [45,122,123]. When drugs are encapsulated within CSNPs, they can be effectively eliminated through urine, resulting in faster drug excretion. This helps to prevent drug accumulation in the body, reducing the risk of toxicity and maintaining optimal drug levels, thereby improving drug activity [101].

Various formulations and strategies have been developed to enhance the oral bioavailability of phytochemicals, which are bioactive compounds derived from plants with potential therapeutic benefits (Table 1). The objective of these formulations is to improve the stability, solubility, mucoadhesive properties, and overall bioavailability of the phytochemicals. Similarly, CSNPs provide efficient delivery of phytochemicals, by resulting in improved stability, controlled release, and increased cellular uptake. Pharmacokinetic studies, biodistribution analyses, and efficacy assessments in animal models demonstrate promising outcomes, including increased absorption, improved bioavailability, sustained release, and enhanced therapeutic effects of phytochemicals. These advancements hold significant potential for the development of effective phytochemical-based therapies.

Sonin et al. researched the biological safety and biodistribution of chitosan nanoparticles, specifically focusing on their intravenous administration. The results indicated that an unmodified chitosan suspension was well-tolerated within the tested dosage range [128]. Various factors, including size, surface charge, administration route, and targeted delivery, influence the biodistribution of the chitosan nanoparticles. Additionally, the shape of the particles plays a significant role in determining their transportation and distribution within the body, resulting in distinct accumulation patterns in different organs [129].

To enhance the targeting capabilities of chitosan nanoparticles, researchers have explored strategies such as passive targeting, molecular targeting, and magnetic targeting. These approaches aim to guide the nanoparticles to accumulate at specific target sites [130]. Chitosan particles exhibit rapid distribution and clearance, especially after intravenous administration. Modifications of chitosan can alter its biological and toxicological profile, allowing for targeted and triggered release from chitosan-based nanoparticles. Typically, chitosan nanoparticles tend to accumulate in the liver, spleen, lungs, and kidneys following systemic administration [131]. Both low- and high-molecular-weight CS will undergo metabolism as well as enzymatic degradation within the body, leading to removal by renal clearance [132].

## 4. Pharmacological Enhancement of Antioxidant and Anticancer Activity in Chitosan-Based Nanoparticles

Using CSNPs can improve the oral delivery of phytochemicals by enhancing their absorption and bioavailability in the intestines. CSNPs overcome challenges such as poor solubility, metabolism, and degradation, resulting in better availability of phytochemicals. Animal studies have shown that CSNPs effectively increase the bioactivity of phytochemicals. By encapsulating them in CSNPs, advantages such as improved solubility, stability in the GI tract, controlled release, and enhanced absorption are achieved, leading to better oral availability. This opens up opportunities for oral phytochemical administration in various treatments. However, it is important to optimize the preparation method of CSNPs, considering factors such as size, shape, and charge, for the desired effects.

### 4.1. Antioxidant Activity

CSNPs have emerged as a promising approach to enhancing the antioxidant activity of bioactive compounds. These NPs offer precise control over size, surface charge, and stability, enabling improved interactions with ROS and free radicals. By effectively scavenging and neutralizing these harmful species, CSNPs reduce oxidative stress and protect cells from damage. The encapsulation of antioxidant compounds within CSNPs enhances stability and controlled release, prolonging their antioxidant effects. Functionalization and surface modifications further enhance their antioxidant properties, allowing targeted delivery to sites of oxidative stress.

CSNPs exhibit enhanced antioxidant activity due to their unique physicochemical properties (Table 2). Derived from chitin, CS contains amino and hydroxyl groups that possess inherent antioxidant properties [133]. These functional groups facilitate electron transfer reactions, enabling CSNPs to scavenge and neutralize ROS and free radicals. The high surface-area-to-volume ratio of these NPs promotes increased contact with reactive species, further enhancing their antioxidant activity. Precise control over particle size and surface charge during preparation allows for optimal bioavailability and cellular uptake. This control is crucial in targeting specific sites of oxidative stress. With their intrinsic antioxidant properties and customizable preparation methods, CSNPs provide a versatile platform for delivering antioxidants and combating oxidative-stress-related diseases.

Studies have suggested that EGCG may help to reduce the risk of breast cancer and may also improve the effectiveness of chemotherapy and radiation therapy.

### 4.2. Anticancer Activity

CSNPs show great promise in improving the delivery of anticancer drugs. By optimizing their size and surface properties, CSNPs can efficiently penetrate tumor tissues and accumulate drugs at the intended site. To enhance their targeting ability, CSNPs can be modified with ligands such as antibodies or peptides, enabling active targeting of cancer cells [36,100].

The preparation method of CSNPs offers versatility in incorporating various types of anticancer agents, including chemotherapeutic drugs, nucleic acids, and phytochemicals. The encapsulation of these agents within CSNPs brings several advantages. Firstly, it enhances their stability, protecting them from degradation and maintaining their efficacy over time. Secondly, it facilitates controlled release, ensuring a sustained and targeted delivery of the therapeutic agents. Additionally, the mucoadhesive properties of CSNPs enable prolonged contact with cancer cells, leading to increased uptake of the encapsulated agents and thereby enhancing their effectiveness. Moreover, the ability to modify the surface of CSNPs with targeting ligands provides a means for selective delivery to cancer cells. By attaching ligands specific to tumor markers, CSNPs actively target cancer cells, improving the therapeutic index and reducing systemic toxicity associated with conventional treatments. Overall, the systematic approach of utilizing CSNPs offers significant promise for the efficient and targeted delivery of a wide range of anticancer agents. The development of dual drug delivery systems using NPs offers promising prospects for improving the delivery and therapeutic efficacy of anticancer agents. This active targeting approach increases the selectivity of therapeutic agents, promoting their uptake by cancer cells while minimizing harm to healthy tissues.

CSNP-based therapy can be employed to enhance the delivery of anticancer drugs to a broad range of cancer cells, resulting in increased cytotoxicity and improved drug accumulation, selectivity, and effectiveness. CSNPs can incorporate different types of hydrophilic or hydrophobic chemotherapy drugs, nucleic acids, and photosensitizers, which can then be targeted to specific tumor cells through the enhanced permeability and retention (EPR) effect and ligand–receptor interactions. Additionally, the cationic structure of CS enables the efficient release of the encapsulated payloads into the cytoplasm of the nanoparticle endosomes [36].

### 4.3. Antioxidant and Anticancer Activity Enhancement

CSNPs play a significant role in enhancing antioxidant and anticancer activities through various interconnected mechanisms. Firstly, their ability to create a protective environment for encapsulated bioactive compounds ensures the stability and controlled release of therapeutic agents, optimizing their efficacy. Secondly, the unique physicochemical properties of CSNPs, such as their small size and large surface area, enable efficient cellular uptake and improved penetration into tumor tissues. This targeted delivery allows for enhanced interaction with specific intracellular targets, thereby increasing the anticancer activity of the agents. Additionally, the positive surface charge of CSNPs facilitates their internalization into cancer cells through electrostatic interactions with cell membranes, leading to the increased accumulation of therapeutic agents and intensified cytotoxic effects.

Antioxidants and anticancer agents are both substances that can have an impact on the development and progression of cancer. While antioxidants can protect cells from damage caused by free radicals and potentially reduce the risk of certain types of cancer, the relationship between antioxidants and cancer is complex and not fully understood. Similarly, while some anticancer agents may work by inducing oxidative stress and causing damage to cancer cells, others may have antioxidant properties and protect healthy cells.

Furthermore, CSNPs possess intrinsic antioxidant activity due to the presence of CS itself. This enables them to scavenge and neutralize free radicals and ROS. Even when formulated into NPs, the CS structure retains its intrinsic antioxidant activity, providing an additional layer of protection against oxidative stress. Moreover, CSNPs can modulate cellular signaling pathways and gene expression, influencing various molecular targets involved in cancer progression. By inducing apoptosis, inhibiting tumor/growth, and suppressing metastasis, they further enhance their anticancer effects.

By integrating these multifaceted mechanisms, CSNPs serve as effective delivery systems for therapeutic agents, enhancing their antioxidant and anticancer activities (Table 3). Their protective encapsulation, efficient cellular uptake, and intrinsic characteristics provide a systematic approach to improving the efficacy of bioactive compounds in combating oxidative stress and inhibiting cancer cell growth.

## 5. Perspective

CS has emerged as a reliable compound carrier widely utilized for the delivery of hydrophobic drugs, including natural antioxidants [6,89,100]. However, the hydrophobic nature of antioxidants presents inherent challenges regarding their solubility, stability, and bioavailability, as well as drug release and active targeting of specific tissues or cells. To tackle these challenges, extensive research has been conducted to enhance the properties of CS, particularly its aqueous solubility and stability. These improvements in the pharmacokinetic profile of CS have the potential to enhance the pharmacological effects of the delivered compounds. Notably, the incorporation of CSNPs into specialized drug delivery methods and formulations has been a promising strategy to address the issue of poor oral bioavailability. CSNPs demonstrate the ability to enhance the bioavailability of antioxidants, while also providing protection during distribution and enabling targeted delivery to specific sites. The ongoing conceptual advancements and practical research endeavors in this field are driving the development of oral delivery systems that leverage CS and its modifications.

To ensure the commercial viability of phytochemical-loaded CSNPs, it is necessary to conduct clinical trials. These trials involve substantial financial costs for research, development, and marketing. However, they are crucial in establishing the safety and effectiveness of CSNPs. Additionally, it is important to develop simple and scalable techniques for the large-scale production of CSNPs. These techniques should adhere to the “GRAS” (generally recognized as safe) category, ensuring their suitability for widespread use. Clear progress in these areas will enhance the feasibility and market readiness of CSNPs.

Further investigation is required to evaluate the toxicity of different CS derivatives in both their free form and when used to encapsulate phytochemicals. Pre-clinical studies are necessary to assess the safety and efficacy of CS-based nanocarriers. A thorough examination of the immunological profiles of CS and its derivatives is crucial to establish a complete safety profile, as current information is limited. While pre-clinical studies have demonstrated the immune-enhancing properties of CS and CS-derivative-based NPs, there is a lack of clinical data regarding their toxicological aspects. Hence, future research should prioritize clinical investigations to gain a better understanding of immune responses. Some reports suggest that adjusting the deacetylation degree of CS can reduce toxicity, but further pre-clinical and clinical studies are necessary to support this claim. The use of different chemicals in the development of CS-based formulations raises concerns, and large-scale production and scalability pose additional challenges for clinical applications. Future research should focus on feasible pre-clinical and clinical investigations. Despite extensive studies on phytochemical-loaded CSNPs, there are currently no commercially available CSNPs products for the management of chronic diseases.

The stability of nanoparticles can be a common concern due to issues such as:

(a) Agglomeration occurs when nanoparticles aggregate, leading to the loss of their unique properties and reduced stability [142]. Surface modification and the use of stabilizing agents can help to prevent agglomeration by creating repulsive forces between particles [143,144,145]. 

(b) Ostwald ripening, where larger particles grow at the expense of smaller ones [146,147], can be minimized by starting with uniformly sized nanoparticles and optimizing synthesis conditions [148,149,150]. 

(c) The leached substances may interact with biological systems or impact the overall behavior and fate of the nanoparticles [151] but this can be mitigated by material selection and surface passivation [152]. 

(d) Environmental factors such as pH, temperature, and humidity can impact stability, thus, proper storage and designing nanoparticles for environmental compatibility are important. Finally, optimization of the synthesis parameters and post-processing treatments can enhance the stability in different types of nanoparticles [153,154,155].

**Figure 3:**
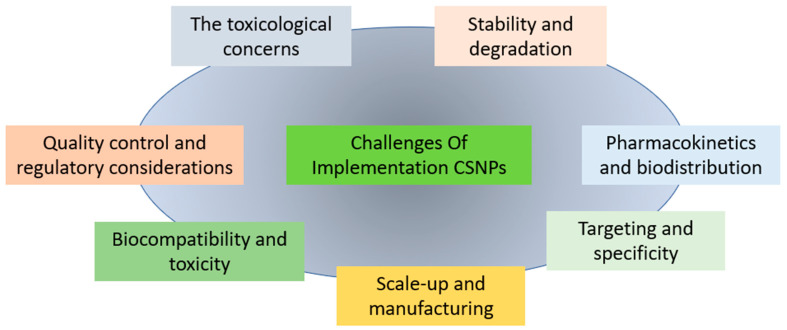
Challenges of the implementation of CSNPs.

Addressing these challenges (Figure 3) through ongoing research and technological advancements will pave the way for the successful application of CSNPs in oral drug delivery, unlocking their full potential for improving drug efficacy, patient compliance, and treatment outcomes.

## 6. Conclusions

In conclusion, CSNPs hold significant promise in oral drug delivery due to their unique properties and versatility. They offer numerous advantages, including enhanced drug solubility, stability, controlled release, and active targeting of specific tissues or cells. By carefully selecting the preparation method, CSNPs can enhance the efficacy of therapeutic agents in various areas, including antioxidant activity, anticancer activity, and pharmacokinetic properties enhancement. However, there are still challenges to overcome, such as stability, scale-up of manufacturing, quality control, pharmacokinetics, biocompatibility, and targeting. By addressing these challenges through continued research and technological advancements, we can harness the full potential of CSNPs in oral drug delivery, leading to improved drug efficacy, patient compliance, and treatment outcomes.

## Figures and Tables

**Table 1:** Various strategies to enhance the oral pharmacokinetic of natural antioxidants.

Natural Antioxidant	Chitosan	Preparation	Characterization of NPs	Pharmacokinetic Enhancement	Pharmacologic Enhancement	Ref.
α-Mangostin	CS and thiolated CS (TCS) are crosslinked using genipin (GP), and the surface is then modified using Eudragit L100	Crosslinked CSNPs	d CSNPs = 437–922 nm, d TCS-based NPs = 365–767 nm, both possessing positive charges on the surface.	TCS-based NPs with GP and L100 exhibit strong mucoadhesion to colon mucosa and provide an increase in α-mangostin loading for controlled-release drug delivery to the colon while limiting its release in the upper GI tract.	The active compound, α-mangostin, was released from NPs and showed effective anti-tumor activity against HT-29 colorectal cancer cells. The combination of pH-dependent and mucoadhesive properties in the TCS NPs allowed for specific delivery of α-mangostin to the colon site, resulting in anti-tumor activity.	[124]
Curcumin (CUR)	N-trimethyl CS/alginate BEADS complexes	Polyelectrolyte complexes (PECs)	The dry samples had an average diameter of around 0.10–0.20 mm. During drying, the surfaces of the beads folded, which created blockages in the material’s pores.	A controlled manner by using beads loaded with CUR as a drug carrier. The results were promising, with 100% of the CUR being released in the simulated intestinal fluid within 24 h.	The biological activity of CUR is enhanced when it is close to the physiological pH.	[125]
Curcumin	N-dodecyl CS-HPTMA chloride-coated liposomal	CS-coated NPs	ZP (31.6–32.3 mV) measurements confirmed the effective coating of liposomes with all these CS derivatives. Particle size 73.15 nm.	These NPs can easily enter the cell membrane and release curcumin in a controlled manner.	Liposomal curcumin showed higher uptake in tumor cells than normal cells, resulting in increased cytotoxicity towards B16F10 cancer cells without significant negative effects on normal cells.	[126]
Quercetin	TPP-chitosomes are a hybrid system consisting of liposomes coated with crosslinked CS.	CS-coated NPs	The nanocarriers were small, spherical particles (~180 nm) with high entrapment efficiency (~91%).	The protective polyelectrolyte shell layer shielded the vesicles and drug from stomach acidity. The system resisted acidity and released in alkaline pH such as the intestines. Quercetin release depended on pH (preferably alkaline) and was controlled by drug diffusion through the hybrid system.		[127]

**Table 2:** Various strategies to enhance the pharmacologic effect of natural antioxidants.

Natural Antioxidant	Chitosan	Preparation	Characterization of NPs	Pharmacologic Effect Enhancement	Ref.
Peppermint oil (PO) and green tea oil (GTO)	CS-PO-NPs/CS-GTO NPs	Ionic gelation method mediated by TPP	The NPs had a spherical shape with an average size range of 20–60 nm (TEM). They exhibited a loading capacity of 22.2% for PO and 23.1% for GTO. The release of drugs in different buffer systems followed a Fickian behavior in vitro.	The NPs significantly improved the antioxidant activity, enhancing it approximately 2-fold for PO and 2.4-fold for GTO.	[134]
Alfa-lipoic acid (ALA)	ALA-CS-GFP-NPs	Ionic gelation in the presence of ALA	d = 44 nm and ZP = +32 mV. They effectively entered 3T3-L1 fibroblasts and crossed the intestinal barrier in vitro. ALA was released slowly from the NPs, indicating their stability in the stomach and subsequent absorption in the intestines.	Encapsulating ALA in CS-ALA-NPs did not alter its antioxidant activity. The CS-ALA-NPs retained their antioxidant activity and remained stable in simulated stomach conditions for up to 3 h.	[135]
Epigallocatechin-3-gallate (EGCG)	EGCG CNPs	Ionic gelation method mediated by TPP	The particle diameters of EGCG CNPs ranged from 41.31 to 388.36 nm.	Even a small concentration of 1.0 µg/mL of EGCG CNPs improved the antioxidant capacity and quality of Kacang buck semen after thawing.	[136]
Aqueous grape extract (AGE)	CS-AGE-NPs	Ionic gelation method mediated by TPP	The size of the NPs was 177.5 ± 2.12 nm, and they had a positive charge of 32.95 ± 0.49 mV. The CSNPs demonstrated good encapsulation efficiency and loading capacity.	The grape extract, when in its free form, exhibited antioxidant activity ranging from 15.6% to 51.01%. However, when the extract was encapsulated, its antioxidant activity increased further, ranging from 21.2% to 62.8%.	[137]
Resveratrol (RES)-loaded protein–polysaccharide NPs	RES–ALA–CSNPs	Oppositely charged α-lactalbumin (ALA) and chitosan (CS) interact through electrostatic forces	d RES–ALA–CHI NPs were 211.0 nm and Z = 13.23 mV.	The interaction between α-lactalbumin (ALA) and CS is based on simple electrostatic interactions between their opposite charges.	[136]

**Table 3:** Various strategies to enhance the pharmacologic effect of phytochemicals.

Natural Antioxidant	Chitosan	Preparation	Characterization of NPs	Antioxidant Enhancement	Anticancer Enhancement	Ref.
Naringenin (NAR)	CSNPs/NAR	Ionic gelation method by tripolyphosphate (TPP).	The native CSNPs had a size of 53.2 nm, which increased to 407.47 nm when loaded with NAR. The encapsulation efficiency of CSNPs/NAR was approximately 70% and 80% (HPLC method). Around 15% of the NAR was released from CSNPs/NAR, suggesting that the CSNPs effectively retained a high amount of NAR in the simulated gastric fluid (SGF), enhancing the drug’s bioavailability.	Using CSNPs/NAR at 0.3 mg/mL and 0.5 mg/mL led to a significant decrease in nitrite levels, reducing nitrate rates by 0.16 M and 0.12 M, respectively. NAR and BHT also showed significant reductions, with rates of 0.17 M and 0.19 M, respectively.	CS-encapsulated NAR was better than using free NAR alone. This highlights an effective system for delivering NAR with antioxidant and anticancer properties.	[41]
Astaxanthin (AST)	CS-AST-NPs	Ionic gelation method by tripolyphosphate (TPP).	d = 505.2 nm in size, Z = +20.4 mV, and showed uniformity in their size distribution. They effectively encapsulated around 63.9% of the drug. These NPs released the drug slowly, allowing it to stay in the bloodstream for a longer time.	The lipid peroxidation and DPPH assay results demonstrate that the ACT-NPs effectively preserved the antioxidant activity of ASX.	The ACT-NPs exhibited enhanced cytoprotective effects on the BHK-21 cell line, providing increased protection to the cells.	[138]
Quercetin (QUE)	CS-QUE-NPs	Ionic gelation method by tripolyphosphate (TPP).	PDI = 0.208, a hydrodynamic d = 103.2 nm, and a positive ZP of +30.4 mV. Quercetin encapsulation efficiency was 83.8%, and its release followed a gradual and faster pattern in pH 7.4 NaH_2_PO_4_ solutions through a non-Fickian mechanism.	The NPs exhibited a stronger antioxidant effect compared to free quercetin.	Quercetin encapsulated in NPs exhibited significant cytotoxicity against MCF-7 (human breast tumor) and A549 (human lung tumor) cells over a 72 h duration.	[139]
Astaxanthin (AXT)	Glycol CS (GC)-decorated AXT NPs (GC-AXT-NPs)	GC and AXT self-organize in water through ionic interactions.	The bioavailability of AXT could be enhanced by formulating AXT NPs that self-organize with GC.	The AXT NPs demonstrated higher inhibition effects on the production of nitric oxide (NO) and the secretion of prostaglandin E2 (PGE2) compared to AXT alone.	The GC-AXT NPs promoted cell migration and proliferation in L292 cells during scratch assays. Additionally, the viability of L929 fibroblast cells remained similar to that of normal cells, indicating no significant changes caused by the NPs.	[140]
Ag-NPs	CS-Ag-NPs composite	The NPs were synthesized using a chemical reduction process, with CS serving as both a reducing agent and a stabilizing agent.	d = 9–65 nm.	The NPs showed high antioxidant activity at different concentrations: 92%, 90%, and 75% at 4000, 2000, and 1000 µg/mL, respectively. The IC_50_ value, representing the concentration needed for 50% inhibition, was 261 µg/mL for Chi/Ag-NPs.	The NPs showed lower toxicity towards normal human skin cell line (BJ-1) cells compared to doxorubicin, which demonstrated higher toxicity.	[141]

## Data Availability

The data that support the findings of this study are available on request from the corresponding author.
